# Public Health Response to a Case of Paralytic Poliomyelitis in an Unvaccinated Person and Detection of Poliovirus in Wastewater — New York, June–August 2022

**DOI:** 10.15585/mmwr.mm7133e2

**Published:** 2022-08-19

**Authors:** Ruth Link-Gelles, Emily Lutterloh, Patricia Schnabel Ruppert, P. Bryon Backenson, Kirsten St. George, Eli S. Rosenberg, Bridget J. Anderson, Meghan Fuschino, Michael Popowich, Chitra Punjabi, Maria Souto, Kevin McKay, Samuel Rulli, Tabassum Insaf, Dustin Hill, Jessica Kumar, Irina Gelman, Jaume Jorba, Terry Fei Fan Ng, Nancy Gerloff, Nina B. Masters, Adriana Lopez, Kathleen Dooling, Shannon Stokley, Sarah Kidd, M. Steven Oberste, Janell Routh, 2022 U.S. Poliovirus Response Team, 2022 U.S. Poliovirus Response Team, Hanen Belgasmi, Barrett Brister, James E. Bullows, Cara C. Burns, Christina J. Castro, Janine Cory, Naomi Dybdahl-Sissoko, Brian D. Emery, Randall English, Ann D. Frolov, Halle Getachew, Elizabeth Henderson, Alexandra Hess, Karen Mason, Jeffrey W. Mercante, Stacey Jeffries Miles, Hongmei Liu, Rachel L. Marine, Nehalraza Momin, Hong Pang, Daniel Perry, Shannon L. Rogers, Brandon Short, Hong Sun, Farrell Tobolowsky, Eileen Yee, Scott Hughes, Enoma Omoregie, Jennifer B. Rosen, Jane R. Zucker, Mohammed Alazawi, Ursula Bauer, Alex Godinez, Brianna Hanson, Eugene Heslin, James McDonald, Neida K. Mita-Mendoza, Megan Meldrum, Dana Neigel, Robin Suitor, David A. Larsen, Christina Egan, Nicola Faraci, G. Stephanie Feumba, Todd Gray, Daryl Lamson, Jennifer Laplante, Kathleen McDonough, Natalie Migliore, Amruta Moghe, Simon Ogbamikael, Jonathan Plitnick, Rama Ramani, Lindsey Rickerman, Erik Rist, Lynsey Schoultz, Matthew Shudt, Julie Krauchuk, Eric Medina, Jacqueline Lawler, Heather Boss,, Emanuele Barca, Danish Ghazali, Tarini Goyal, Sean J.P. Marinelli, Jackson A. Roberts, Grace B. Russo, Kiran T. Thakur, Vivian Q. Yang

**Affiliations:** ^1^2022 CDC Domestic Poliovirus Emergency Response Team; ^2^New York State Department of Health; ^3^Department of Epidemiology and Biostatistics, State University of New York at Albany, Albany, New York; ^4^Rockland County Department of Health, Pomona, New York; ^5^Wadsworth Center, New York State Department of Health; ^6^Department of Biomedical Science, State University of New York at Albany, Albany, New York; ^7^Department of Public Health, Syracuse University, Syracuse, New York; ^8^Orange County Department of Health, Goshen, New York.; CDC; CDC; CDC; CDC; CDC; CDC; CDC; CDC; CDC; CDC; CDC; CDC; CDC; CDC; CDC; CDC; CDC; CDC; CDC; CDC; CDC; CDC; CDC; CDC; CDC; CDC; New York City Department of Health and Mental Hygiene; New York City Department of Health and Mental Hygiene; New York City Department of Health and Mental Hygiene; CDC and New York City Department of Health and Mental Hygiene; New York State Department of Health; New York State Department of Health; New York State Department of Health; New York State Department of Health; New York State Department of Health; New York State Department of Health; New York State Department of Health; New York State Department of Health; New York State Department of Health; New York State Department of Health; Syracuse University; Wadsworth Center, New York State Department of Health; Wadsworth Center, New York State Department of Health; Wadsworth Center, New York State Department of Health; Wadsworth Center, New York State Department of Health; Wadsworth Center, New York State Department of Health; Wadsworth Center, New York State Department of Health; Wadsworth Center, New York State Department of Health; Wadsworth Center, New York State Department of Health; Wadsworth Center, New York State Department of Health; Wadsworth Center, New York State Department of Health; Wadsworth Center, New York State Department of Health; Wadsworth Center, New York State Department of Health; Wadsworth Center, New York State Department of Health; Wadsworth Center, New York State Department of Health; Wadsworth Center, New York State Department of Health; Wadsworth Center, New York State Department of Health; Rockland County Department of Health, New York; Rockland County Department of Health, New York; Orange County Department of Health, New York; Orange County Department of Health, New York; Columbia University Irving Medical Center-New York Presbyterian Hospital; Columbia University Irving Medical Center-New York Presbyterian Hospital; Columbia University Irving Medical Center-New York Presbyterian Hospital; Columbia University Irving Medical Center-New York Presbyterian Hospital; Columbia University Irving Medical Center-New York Presbyterian Hospital; Columbia University Irving Medical Center-New York Presbyterian Hospital; Columbia University Irving Medical Center-New York Presbyterian Hospital; Columbia University Irving Medical Center-New York Presbyterian Hospital

On July 18, 2022, the New York State Department of Health (NYSDOH) notified CDC of detection of poliovirus type 2 in stool specimens from an unvaccinated immunocompetent young adult from Rockland County, New York, who was experiencing acute flaccid weakness. The patient initially experienced fever, neck stiffness, gastrointestinal symptoms, and limb weakness. The patient was hospitalized with possible acute flaccid myelitis (AFM). Vaccine-derived poliovirus type 2 (VDPV2) was detected in stool specimens obtained on days 11 and 12 after initial symptom onset. To date, related Sabin-like type 2 polioviruses have been detected in wastewater* in the patient’s county of residence and in neighboring Orange County up to 25 days before (from samples originally collected for SARS-CoV-2 wastewater monitoring) and 41 days after the patient’s symptom onset. The last U.S. case of polio caused by wild poliovirus occurred in 1979, and the World Health Organization Region of the Americas was declared polio-free in 1994. This report describes the second identification of community transmission of poliovirus in the United States since 1979; the previous instance, in 2005, was a type 1 VDPV (*1*). The occurrence of this case, combined with the identification of poliovirus in wastewater in neighboring Orange County, underscores the importance of maintaining high vaccination coverage to prevent paralytic polio in persons of all ages.

## Case Findings

In June 2022, a young adult with a 5-day history of low-grade fever, neck stiffness, back and abdominal pain, constipation, and 2 days of bilateral lower extremity weakness visited an emergency department and was subsequently hospitalized with suspected AFM; the patient was unvaccinated against polio (Figure). As part of national AFM surveillance,^†^ the suspected case was reported to NYSDOH and then to CDC. The patient was discharged to a rehabilitation facility 16 days after symptom onset with ongoing lower extremity flaccid weakness. A combined nasopharyngeal/oropharyngeal swab and cerebrospinal fluid sample were negative by reverse transcription–polymerase chain reaction (RT-PCR) testing for enteroviruses and human parechovirus, as well as for a panel of common respiratory pathogens and encephalitic viruses by molecular methods (*2*). RT-PCR and sequencing of a stool specimen by the NYSDOH laboratory identified poliovirus type 2. Specimens were tested at CDC using RT-PCR (*3*) and sequencing, confirming the presence of poliovirus type 2 in both stool specimens. Additional sequencing identified the virus as VDPV2 (*4*), differing from the Sabin 2 vaccine strain by 10 nucleotide changes in the region encoding the viral capsid protein, VP1, suggesting transmission for up to 1 year although the location of that transmission is unknown.

**FIGURE:**
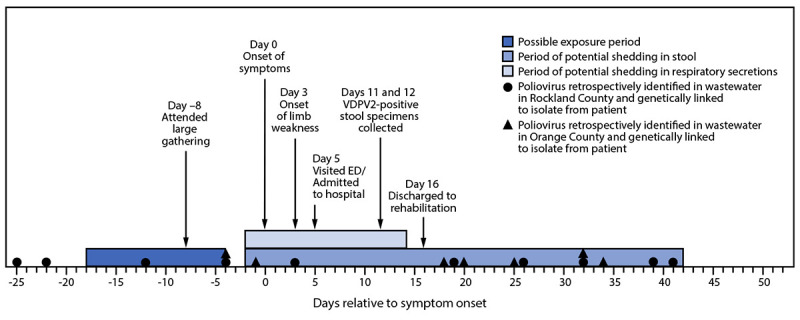
Timeline of patient activities, potential poliovirus exposures, shedding, and poliovirus-positive wastewater* samples† genetically linked to a patient with a case of type 2 vaccine-derived poliovirus — New York, May–August 2022 **Abbreviations:** ED = emergency department; VDPV2 = type 2 vaccine-derived poliovirus. * Wastewater, also referred to as sewage, includes water from household or building use (e.g., toilets, showers, and sinks) that can contain human fecal waste and water from non-household sources (e.g., rain and industrial use). ^†^ More than one positive wastewater sample might have been collected on the same day in Rockland County or Orange County.

Based on the typical incubation period for paralytic polio, the presumed period of exposure occurred 7 to 21 days before the onset of paralysis.^§^ Epidemiologic investigation revealed that the patient attended a large gathering 8 days before symptom onset and had not traveled internationally during the presumed exposure period. No other notable or known potential exposures were identified.

## Public Health Response

Upon notification of the poliovirus-positive specimen, CDC, NYSDOH, and local health authorities launched an investigation and response on July 18, 2022. Activities included issuing a NYSDOH advisory on July 22 to increase health care provider awareness,^¶^ enhancing surveillance for potentially infected persons, testing wastewater from Rockland and surrounding New York counties, assessing vaccination coverage in the patient’s community, supplying inactivated polio vaccine (IPV) to county immunization providers, and launching vaccination clinics throughout Rockland County.

Enhanced surveillance defined persons under investigation (PUIs) as those who met clinical criteria and who lived in or traveled to specific counties or neighborhoods in New York or had international travel since May 1, 2022.** As of August 10, three additional persons have been classified as PUIs; available specimens from the PUIs (i.e., stool, cerebrospinal fluid, serum, nasopharyngeal, or oropharyngeal swabs) yielded negative poliovirus test results.

As of August 10, a total of 260 wastewater samples from treatment plants in Rockland and Orange Counties, including samples originally collected for SARS-CoV-2 surveillance, were tested for poliovirus. Among these samples, 21 (8%) yielded positive poliovirus test results using RT-PCR and partial genome sequencing, including 13 from Rockland County and eight from Orange County. Twenty specimens from wastewater samples collected during May, June, and July were genetically linked to virus from the patient’s stool samples; one additional sample, from April in Orange County, was sequenced as poliovirus type 2, but the sequence was incomplete, precluding assessment of genetic linkage to the case. After these results, in August 2022, additional clinical and public health surveillance activities, including additional outreach to local providers and syndromic surveillance, were launched to identify the presence of symptomatic nonparalytic infection (characterized by mild symptoms [e.g., low-grade fever and sore throat] or more severe symptoms [e.g., aseptic meningitis])^††^ and asymptomatic infection in the counties with poliovirus-positive wastewater findings.

According to the New York State Immunization Information System, 3-dose polio vaccination coverage among infants and children aged <24 months living in Rockland County was 67.0% in July 2020 and declined to 60.3% by August 2022, with zip code–specific coverage as low as 37.3%.^§§^ National coverage for IPV by age 24 months was 92.7% among infants born during 2017–2018 (*5*). The Rockland County Department of Health launched a countywide catch-up vaccination effort on July 22, 2022. Although there was a brief increase in administration of polio-containing vaccines (IPV alone and combination vaccines including IPV), the number of doses administered at temporary and established clinics was not sufficient to meaningfully increase population IPV coverage levels.

## Discussion

The findings in this report represent only the second community transmission of poliovirus identified in the United States since 1979 (*1*). At present, the origin of the VDPV2 detected in the patient’s stool and in sewage samples remains unknown. Because the patient had not traveled internationally during the potential exposure period, detection of VDPV2 in the patient’s stool samples indicates a chain of transmission within the United States originating with a person who received a type 2-containing oral polio vaccine (OPV) abroad; OPV was removed from the routine immunization schedule in the United States in 2000. Genome sequence comparisons have identified a link to vaccine-related type 2 polioviruses recently detected in wastewater in Israel and the United Kingdom.^¶¶^ In general, approximately one in 1,900 poliovirus type 2 infections among unvaccinated persons is expected to result in paralysis (*6*). As of August 10, 2022, no additional poliomyelitis cases have been identified, although the detection of VDPV2 genetically linked to virus from the patient in wastewater specimens from two counties in New York State over the course of ≥2 months indicates community transmission and ongoing risk for paralysis to unvaccinated persons.

VDPVs can emerge when live, attenuated OPV is administered in a community with low vaccination coverage. Replication of OPV in a person who was recently vaccinated can result in viral reversion to neurovirulence, which can cause paralytic poliomyelitis in unvaccinated persons who are exposed to the vaccine-derived virus. Since removal of OPV from the routine U.S. immunization schedule in 2000, IPV has been the only polio vaccine used in the United States. An inactivated vaccine, IPV does not replicate, revert to VDPV, or cause vaccine-associated paralytic polio. Vaccination with 3 doses of IPV is >99% effective in preventing paralysis***; however, IPV does not prevent intestinal infection and therefore does not prevent poliovirus transmission.

Before this case, the last detection of poliovirus in a person in the United States was in 2013, in an immunocompromised infant who received OPV in India and then immigrated to the United States (*1*). VDPVs were identified in the United States in 2005 and 2008 in unvaccinated or immunodeficient persons who were in contact with a person who had recently received OPV; the 2008 case did not result in community transmission. Globally, type 2-containing vaccine (OPV2) has not been used in routine immunization since 2016, although monovalent OPV2 is used for specific vaccination campaigns to control circulating VDPV2 outbreaks (*7*).

Low vaccination coverage in the patient’s county of residence indicates that the community is at risk for additional cases of paralytic polio. Even a single case of paralytic polio represents a public health emergency in the United States. Vaccination plays a critical role in protecting persons from paralysis if they are exposed to poliovirus. During the COVID-19 pandemic, routine vaccination services were disrupted, leading to a decline in vaccine administration and coverage (*8**,**9*), including with IPV, and leaving many communities at risk for outbreaks of vaccine-preventable diseases. Until poliovirus eradication is achieved worldwide, importations of both wild polioviruses and VDPVs into the United States are possible. This case highlights the risk for paralytic disease among unvaccinated persons; all persons in the United States should stay up to date on recommended IPV vaccination to prevent paralytic disease.^†††^

**Summary**

**What is already known about this topic?**

Sustained poliovirus transmission has been eliminated from the United States for approximately 40 years; vaccines are highly effective in preventing paralysis after exposure.

**What is added by this report?**

In June 2022, poliovirus was confirmed in an unvaccinated immunocompetent adult resident of New York hospitalized with flaccid lower limb weakness. Vaccine-derived poliovirus type 2 was isolated from the patient and identified from wastewater samples in two neighboring New York counties.

**What are the implications for public health practice?**

Unvaccinated persons in the United States remain at risk for paralytic poliomyelitis if they are exposed to either wild or vaccine-derived poliovirus; all persons in the United States should stay up to date on recommended poliovirus vaccination.

## Footnotes

* Wastewater, also referred to as sewage, includes water from household or building use (e.g., toilets, showers, and sinks) that can contain human fecal waste and water from non-household sources (e.g., rain and industrial use). https://www.cdc.gov/healthywater/surveillance/wastewater-surveillance/wastewater-surveillance.html#how-wastewater-surveillance-works

†
https://www.cdc.gov/acute-flaccid-myelitis/hcp/case-definitions.html

§
https://www.cdc.gov/vaccines/pubs/pinkbook/polio.html

¶
https://health.ny.gov/diseases/communicable/polio/docs/2022-07-29_han.pdf

** The full case definition included epidemiologic, clinical, and laboratory criteria. Epidemiologic criteria included being a person who lived in or traveled to specific counties or neighborhoods in the state of New York or traveled internationally since May 1, 2022. Clinical criteria included 1) acute onset of flaccid paralysis of one or more limbs with decreased or absent tendon reflexes in the affected limbs, without other apparent cause, and without sensory or cognitive loss, or 2) meningitis, with either a positive enterovirus test result in any specimen or, if adequate testing for enteroviruses was not available, the absence of another apparent cause. Laboratory criteria included detection of wild or vaccine-derived poliovirus in a clinical specimen. PUIs were persons who met both epidemiologic and clinical criteria; confirmed cases of paralytic polio were defined as meeting both laboratory criteria and clinical criterion 1. Confirmed nonparalytic polio cases were defined as meeting laboratory criteria and clinical criterion 2, or meeting laboratory but not clinical criteria.

††
https://www.cdc.gov/vaccines/pubs/pinkbook/polio.html

§§
https://www.cdc.gov/vaccines/imz-managers/coverage/schoolvaxview/data-reports/index.html

¶¶
https://polioeradication.org/news-post/vaccine-derived-poliovirus-type-2-vdpv2-detected-in-environmental-samples-in-london-uk/

***
https://www.cdc.gov/vaccines/pubs/pinkbook/polio.html

†††
https://www.cdc.gov/vaccines/vpd/polio/public/index.html
